# Pulmonary Carcinosarcoma: A Case Report of Biphasic Lung Tumor

**DOI:** 10.7759/cureus.5643

**Published:** 2019-09-13

**Authors:** Pooja Devi, Natasha Singh, Mathew J Tortora

**Affiliations:** 1 Pathology, Saint Barnabas Medical Center, Robert Wood Johnson Barnabas Health, Livingston, USA

**Keywords:** pulmonary carcinosarcoma, pcs, sarcomatous components

## Abstract

Pulmonary carcinosarcoma is an unusual biphasic tumor of the lung with carcinomatous and sarcomatous components. We report a case in a 71-year-old female who presented with a 13-cm lung mass. Microscopic examination revealed squamous cell carcinoma and chondrosarcoma with focal spindle cell atypia. In the most recent version of the World Health Organization (WHO) classification, carcinosarcoma is included in the category of sarcomatous neoplasms with a poorer prognosis than non-small cell lung carcinoma.

## Introduction

A pulmonary carcinosarcoma (mixed malignant tumor) is an unusual malignant neoplasm of the lung that has poorly differentiated carcinoma and sarcomatous elements [1-2]. Carcinosarcomas are more frequently described in the uterus, esophagus, skin, lungs, and hypopharynx [3]. Pulmonary carcinosarcoma accounts for 0.3% to 1% of all pulmonary neoplasms. It was first described by Kika et al. in 1908 [2-3]. Since the initial description by Kika, more cases have been published with different clinical and histological characteristics. Pulmonary carcinosarcomas are more common in men and strongly associated with smoking and asbestosis.

## Case presentation

A 71-year-old nonsmoker female presented to the primary care physician with the chief complaints of difficulty in breathing, fatigue, and dry cough of eight to nine months duration. The patient had no significant past medical or surgical history. No known family history of cancer was present. She had a chest X-ray followed by computed tomography (CT), which showed a peripheral left lower lobe lung mass with mediastinal and hilar adenopathy. The patient underwent CT-guided lung biopsy of the lesion and an outside biopsy reported poorly differentiated carcinoma. The patient underwent the staging procedure with positron emission tomography-computed tomography (PET-CT) and magnetic resonance imaging (MRI) brain, which revealed no evidence of metastatic disease, but a large fluorodeoxyglucose (FDG)-avid mass in the left lower lobe consistent with clinical T3No lung cancer. She was then referred to our hospital for further care.

**Figure 1:**
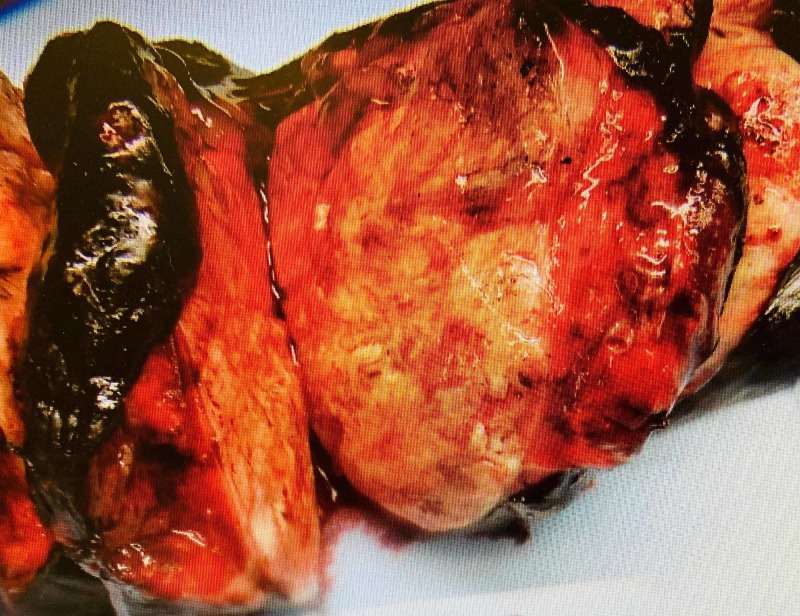
Gross examination of a lobectomy specimen showing mass lesion.

**Figure 2:**
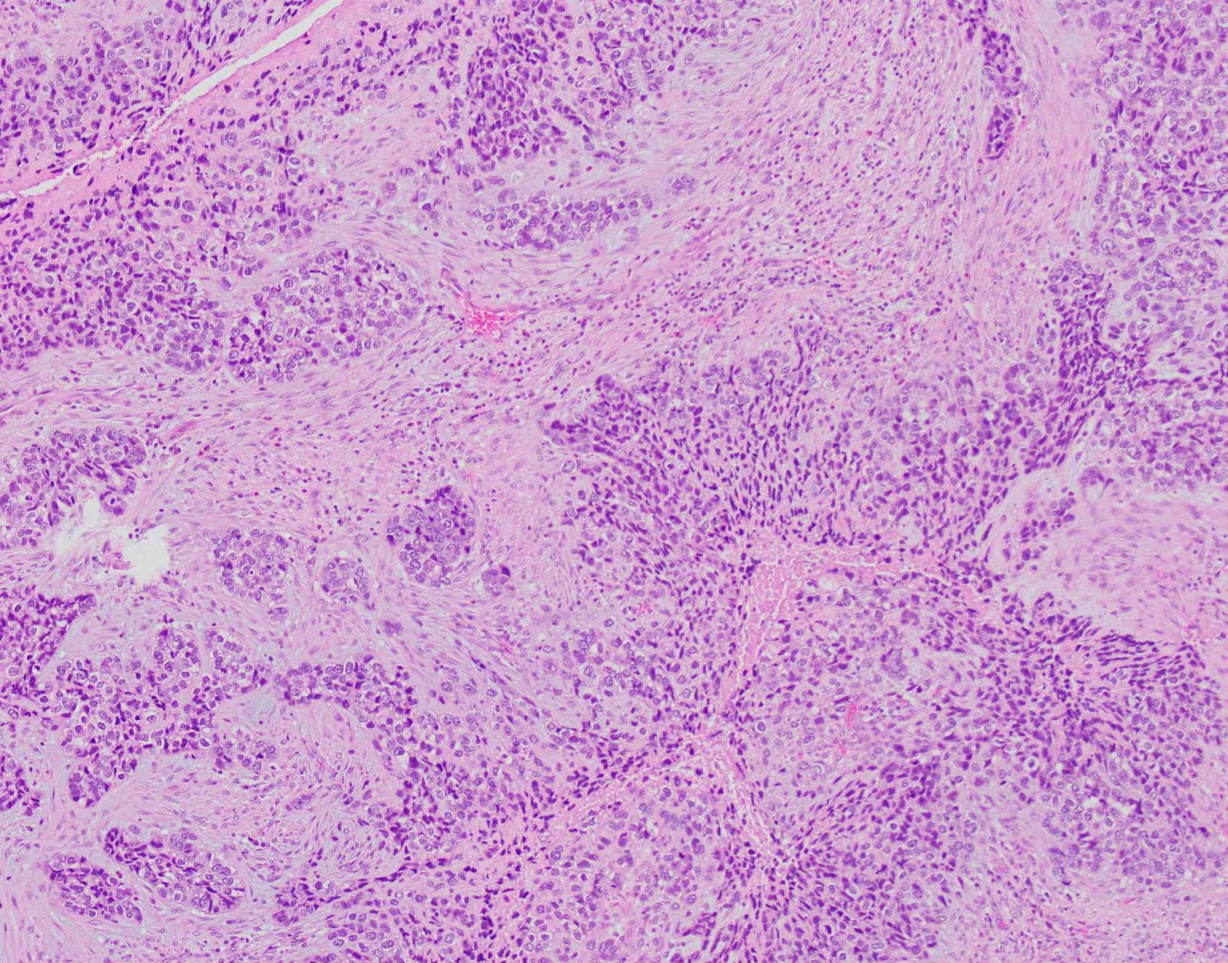
Poorly differentiated squamous cell carcinoma, an epithelial component of the tumor.

**Figure 3:**
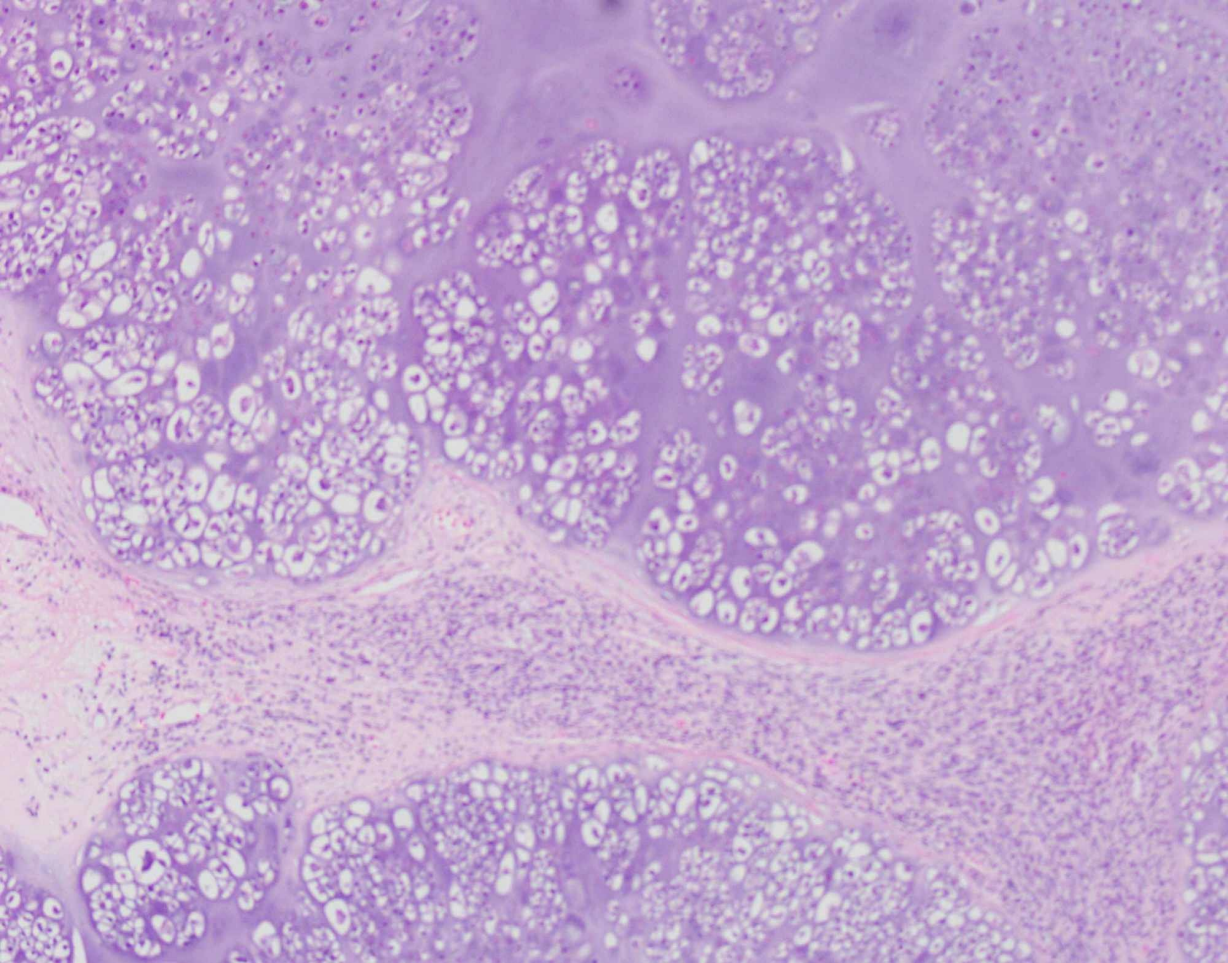
Chondrosarcoma and spindle tumor cells, a mesenchymal component of the tumor.

**Figure 4:**
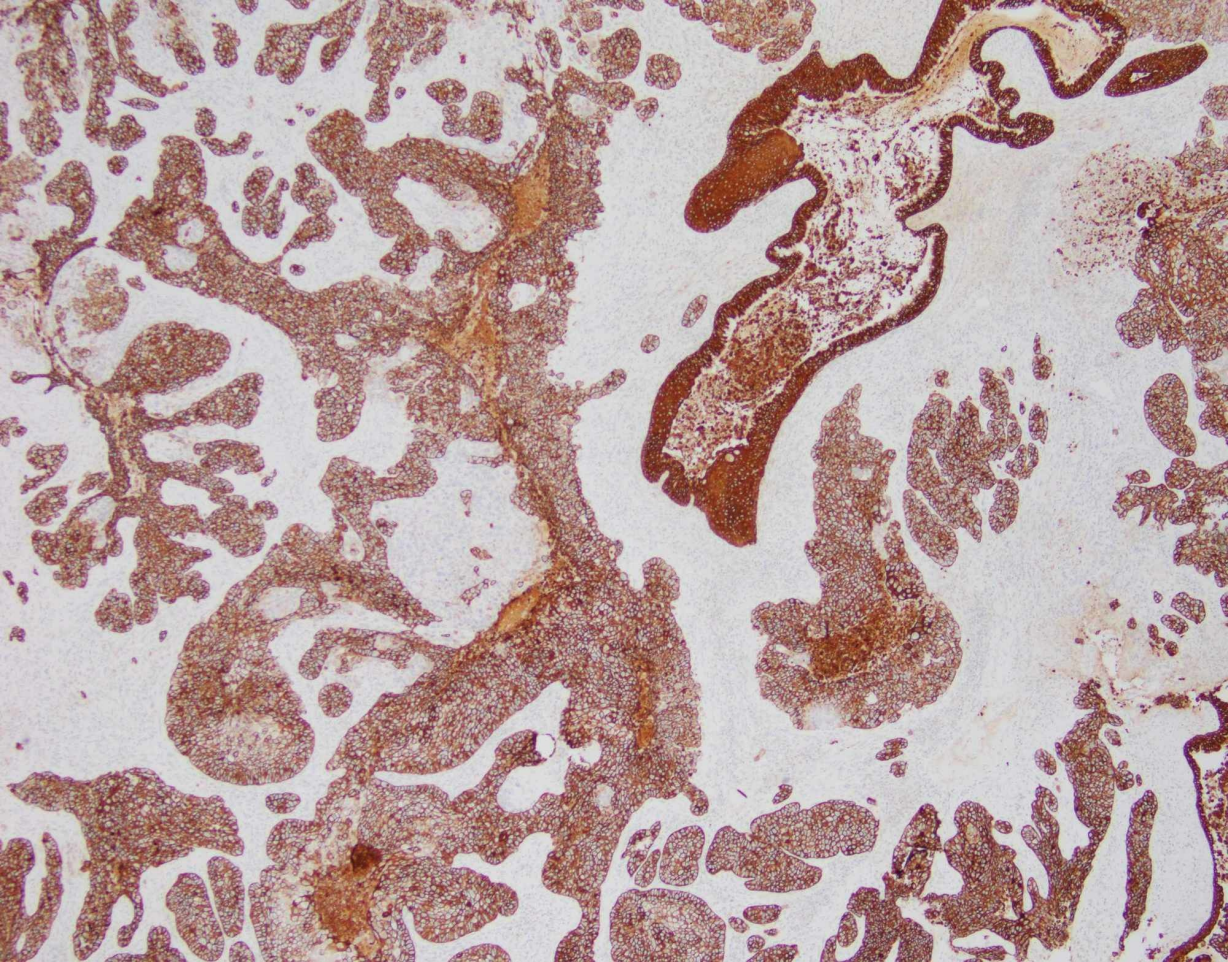
Epithelial component of the tumor positive for cytokeratin (AE1/AE3) immunostain.

**Figure 5:**
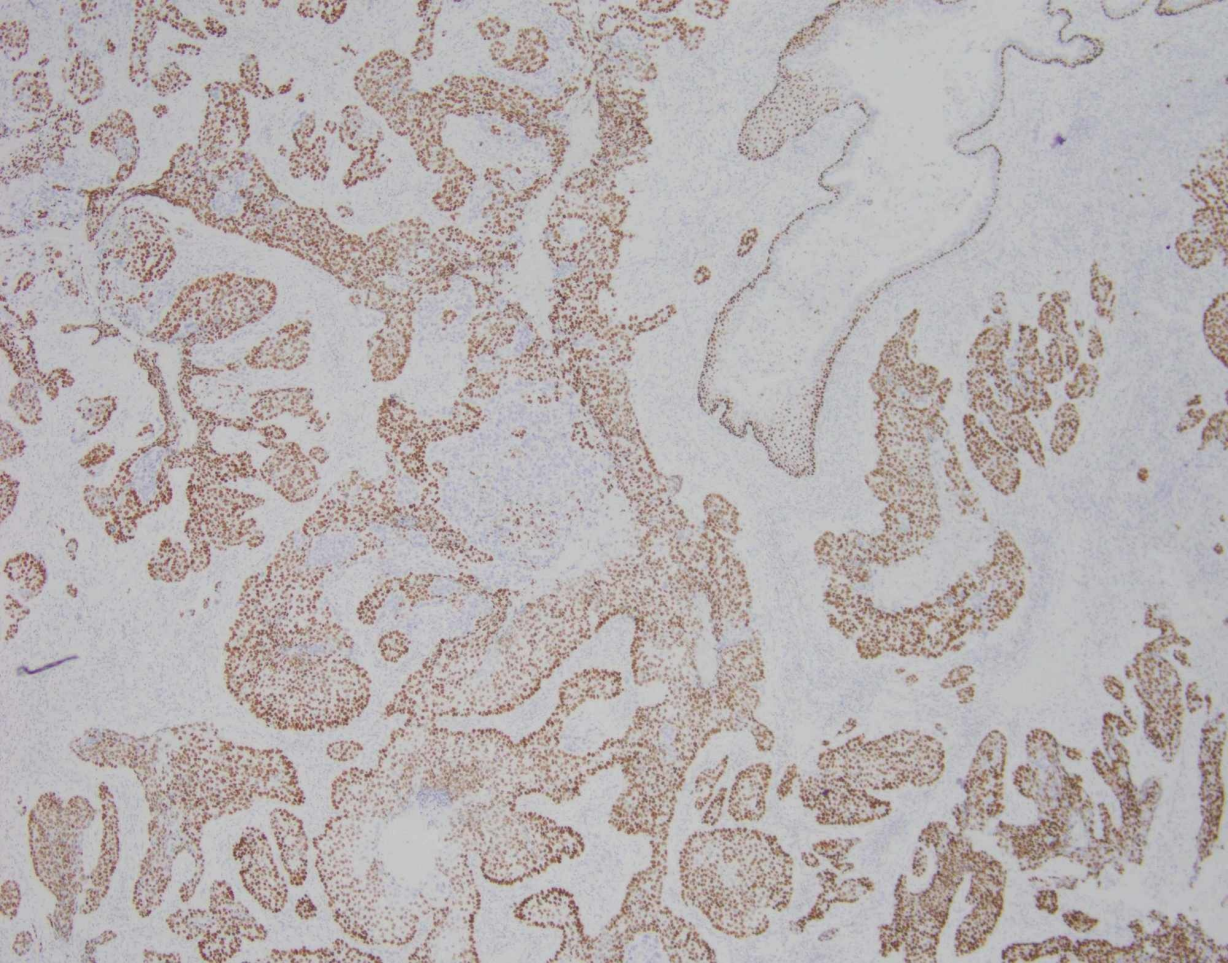
Squamous cell carcinoma component of the tumor positive for the p40 immunostain.

The patient underwent left lower lobectomy and the staging procedure. The lobectomy specimen revealed a grey-white, well-circumscribed, firm mass measuring 13 x 12 x 5cm (Figure 1). The mass protruded into the bronchial lumen with retraction of the overlying pleura. The margins, pleura, and lymph nodes submitted for frozen section analysis were negative for malignancy. Extensive sampling of the tumor was done for permanent microscopic examination. The microscopic examination revealed a malignant neoplasm with epithelial and mesenchymal components. The carcinomatous component showed poorly differentiated squamous cell carcinoma while the sarcomatous component showed predominantly chondroid differentiation with foci of spindle cells with marked atypia and conspicuous mitosis (Figures 2-3). The differential included poorly differentiated non-small cell lung carcinoma with sarcomatoid growth and pulmonary carcinosarcoma. We performed immunohistochemical stains for further evaluation. The epithelial component was positive for cytokeratin (AE1AE3) and p40 (Figures 4-5) while TTF-1 showed patchy positivity within the mesenchymal component and napsin A was negative. The findings were consistent with a pulmonary carcinosarcoma with the pathological staging of pT4 pN0. The patient received eight cycles of adjuvant chemotherapy with gemcitabine and cisplatin. The recent follow-up CT scan was negative for recurrence or distant metastasis.

## Discussion

In 2004, according to the World Health Organization (WHO) classification of lung tumors, sarcomatoid carcinomas are classified into five subtypes: pleomorphic carcinoma, spindle cell carcinoma, giant cell carcinoma, carcinosarcoma, and pulmonary blastoma [1]. Pulmonary carcinosarcoma (PCS) is a poorly differentiated non-small cell carcinoma with carcinomatous and sarcomatoid differentiation [1-2]. The average age of presentation is 60 years, with a male-to-female ratio of 4:1. Clinically, three major forms of pulmonary carcinosarcomas are known: (a) solely endobronchial; (b) solely peripheral or parenchymatous; (c) endobronchial with parenchymatous components [3].

Carcinosarcoma of the lung can cause chest pain, breathing difficulties, fatigue, and other general signs and symptoms, such as fever, weight loss, and appetite loss. Our patient had clinical symptoms of breathing difficulty, fatigue, and dry cough. Radiographic findings comprise a solitary mass, as found in our case, or opacity leading to obstruction and associated pleuroparenchymal changes [3].

The gross features, such as color, size, and consistency, are nonspecific and differ. In a review of 30 cases of carcinosarcoma reported in the literature, all extra-bronchial masses were moderately firm, homogenous, and multinodular and the intrabronchial lesions were polypoid and soft [2-3].

Microscopically, the most common carcinomatous element is squamous cell carcinoma followed by adenocarcinoma and the most common sarcomatous element is the spindle cell elements or fibrosarcoma followed by chondrosarcoma, osteosarcoma, and rhabdomyosarcoma [4-5]. The sarcomatous component may predominate and obscure the carcinomatous component. Therefore, extensive sampling is recommended, and it is suggested to submit one section per centimeter of maximum tumor diameter along with areas of hemorrhage and necrosis. The immunohistochemical stains used for epithelial elements are cytokeratin, TTF1, Napsin, P40, and p63, which is variable based on the morphology of the epithelial component; the sarcomatous component is negative for keratin; other markers are positive according to the differentiation: rhabdomyosarcoma: immunoreactive with desmin, myogenin, and MyoD1, chondrosarcoma: immunoreactive with S100, osteosarcoma: osteocalcin stains the osteoid matrix [6].

The differential diagnoses are relatively few but, based on the predominant component and morphology, include other subtypes of sarcomatoid carcinoma, carcinoma with desmoplastic stroma, malignant mesothelioma, and metastatic sarcoma [1].

Standard therapy for PCS has not yet been established owing to the low incidence of this tumor. Complete surgical resection is the first choice for PCS, if possible. Effective systemic chemotherapy is considered in inoperable patients.

The prognosis of the endobronchial carcinosarcoma is more favorable than that for the peripheral carcinosarcoma, which leads to aggressive outcomes with early metastasis [5]. Metastasis is common to the lymph node, followed by the brain, bone, kidney, and liver. Overall, there is a worse prognosis than for conventional non-small cell carcinoma due to the high tendency for distant metastasis, particularly when the sarcomatous component is predominant [7].

## Conclusions

Pulmonary carcinosarcoma (PCS) is a rare biphasic lung tumor that presents either as a solid mass involving the peripheral lung parenchyma or a polypoid lesion involving the endobronchial tree. It is important to identify the epithelial and mesenchymal components to make the diagnosis. Histopathologic examination and immunohistochemical staining can help to distinguish it from other carcinomatous and sarcomatous neoplasms.
